# What the PHIRI federated research infrastructure has achieved so far?

**DOI:** 10.1093/eurpub/ckac129.467

**Published:** 2022-10-25

**Authors:** J Gonzalez-Garcia

**Affiliations:** Biocomputing Unit, Institute for Health Sciences in Aragon, Zaragoza, Spain

## Abstract

PHIRI infrastructure follows a federated approach that is governed following the European Interoperability Framework. The vision of PHIRI is to create an infrastructure for individual level data processing following the privacy-by-design principle in a data-centric approach. As a basis to legal interoperability and compliance with the GDPR, the queries or algorithms are moved to the data instead of moving the data. So far, the PHIRI technological developments have focused on a client-server architecture. In this architecture a Coordinator Hub, the server, is in charge of orchestrating the deployment of the data-centric analysis solutions, in the form of R and Python scripts, that will be later executed in the partner nodes (data hubs), the clients. To perform the orchestration the Coordinator Hub encapsulates the scripts in software containers, using Docker images; all the outputs are published in Zenodo. The software containers are then deployed manually from Zenodo in the partner nodes and executed by its IT specialists using their own individual level data - the software containers have represented the technical interoperability layer. The data used on each partner has been previously adapted to a common data model (CDM) and the quality of the dataset has been assessed against the data model by each partner -this has represented the semantic interoperability layer. Finally, the outputs of the analysis's execution are aggregated data that are sent back to the Coordinator Hub to perform a comparative analysis. This stepwise approach has been tested in various research questions promoted by a leading researcher and agreed by the partner nodes who act as data hubs. A help-desk services and a developer's forum and a help-desk service have been set up to ease the implementation and deployment of the research queries - these both have represented the organisational interoperability layer

